# N-Acetylcysteine Therapy in Thrombotic Thrombocytopenic Purpura: A Systematic Review and Critical Appraisal

**DOI:** 10.3390/jcm15072713

**Published:** 2026-04-03

**Authors:** Ufuk Demirci, Zübeyir Talha Bilgin, Mehmet Baysal

**Affiliations:** 1 Department of Internal Medicine, Division of Haematology, Medical Faculty, Manisa Celal Bayar University, 45030 Manisa, Türkiye; 2 Department of Internal Medicine, Medical Faculty, Manisa Celal Bayar University, 45030 Manisa, Türkiye; dr.talhabilgin@gmail.com; 3 Department of Internal Medicine, Division of Haematology, Medical Faculty, Namık Kemal University, 59030 Tekirdağ, Türkiye; drmehmetbaysal@gmail.com

**Keywords:** thrombotic thrombocytopenic purpura, N-acetylcysteine, ADAMTS13

## Abstract

**Background:** Thrombotic thrombocytopenic purpura (TTP) is a life-threatening condition resulting from a disintegrin and metalloproteinase with thrombospondin type 1 motif, member 13 (ADAMTS13) deficiency, leading to the accumulation of ultra-large von Willebrand factor (vWF) multimers and widespread microvascular thrombosis. While therapeutic plasma exchange and immunosuppression have significantly improved response, refractory and relapsed disease are significant challenges. N-acetylcysteine (NAC) has emerged as a biologically plausible adjunctive therapy due to its potential to reduce disulfide bonds in vWF multimers. However, its clinical role is unclear. This systematic review aimed to evaluate the clinical evidence regarding the efficacy and safety of N-acetylcysteine in patients with immune-mediated TTP. **Methods:** We performed a systematic review in accordance with the PRISMA guidelines. PubMed/MEDLINE, Google Scholar, and ClinicalTrials.gov were searched until January 2026. Studies involving patients with immune-mediated TTP treated with NAC were included. Case reports, case series, and observational studies involving patients with immune-mediated TTP treated with NAC were included. Risk of bias was evaluated using adapted quality assessment tools. **Results:** Sixteen studies encompassing 69 patients met the inclusion criteria. Most reports were case reports or small case series; two were larger observational cohorts. NAC was predominantly used as adjunctive therapy in relapsed or refractory TTP. Dose regimens varied. Platelet recovery following NAC was reported within 1–15 days in responding cases. Predominantly positive haematological responses were observed in small series. Significant heterogeneity in patient populations, timing of initiation, concomitant therapies, and outcome reporting limited causal inference. **Conclusions:** The current evidence suggests that NAC has a biologically rational and potentially adjunctive value in TTP, particularly in refractory disease or resource-constrained settings. However, current data are largely heterogeneous and derived from low-level evidence. Well-designed prospective studies and randomized controlled trials are needed to determine whether NAC provides significant clinical benefit beyond standard therapy.

## 1. Introduction

Thrombotic Thrombocytopenic Purpura (TTP) is a life-threatening emergency in haematology [1]. ADAMTS13 protease deficiency prevents cleavage of ultra-large von Willebrand factor (ULVWF) multimers, resulting in widespread microvascular thrombosis, severe thrombocytopenia, and organ ischemia [2]. The underlying mechanisms are clear in theory, yet clinical cases carry greater complexity [3].

Immune TTP demands rapid action in practice. Therapeutic plasma exchange (TPE), corticosteroids, and supportive treatment are essential components of treatment. Rituximab and Caplacizumab increase response rates and reduce the risk of relapse; in refractory patients, bortezomib, cyclophosphamide, and vincristine can also be added to the therapy [1]. Diagnostic delays persist due to ADAMTS13 assays. These assays, often unavailable onsite in many facilities, require shipping to reference labs—often days away. TPE treatment begins empirically, by evaluating the blood smear evidence and PLASMIC score [4]. Initiating treatment prior to enzymatic confirmation, followed by a clinical reassessment once ADAMTS13 results become available, can be a life-saving strategy in the majority of cases [5].

Recent studies show that agents such as Caplacizumab have improved outcomes [6]. However, their high cost and unavailability in regions like ours make them impractical for routine use [7,8]. Furthermore, clinical complexity can lead to some cases being resistant to treatment, and relapse and treatment-resistant status can be observed in 20–50% of cases despite standard treatment. This situation is driving clinicians to seek affordable and accessible adjuncts. According to some in vitro and animal studies, as well as case reports, N-acetylcysteine (NAC) might be a promising option [9]. The mechanism of action of NAC involves breaking the disulfide bonds in ULVWF, thereby reducing the size of the multimers [10]. There are studies showing the effect of NAC on platelet function in cases other than TTP. There is a preclinical study in the literature supporting the inhibition of platelet adhesion due to NAC [11]. Furthermore, it is debated whether its effect on platelet function is associated with increased blood loss and blood product use in the postoperative period [12]. Preclinical data suggest this mechanism of action could synergistically complement current therapies; while TPE removes antibodies and replaces the enzyme, NAC provides an immediate biochemical reduction in the existing microthrombi burden. However, the current literature provides uncertain data about the reliability of using NAC in TTP. In resistant TTP, additional treatment options may be limited for clinicians for certain reasons. The side effect profile and accessibility of these treatments are among the limiting factors. Another limiting factor is the combination of financial toxicity and constrained resources, which further complicates treatment accessibility for patients with refractory disease in clinical settings. Based on this premise, this systematic review aims to provide a new perspective on the place of NAC in TTP by examining the existing literature data.

## 2. Methods

### 2.1. Search Strategy and Data Sources

**Figure 1:**
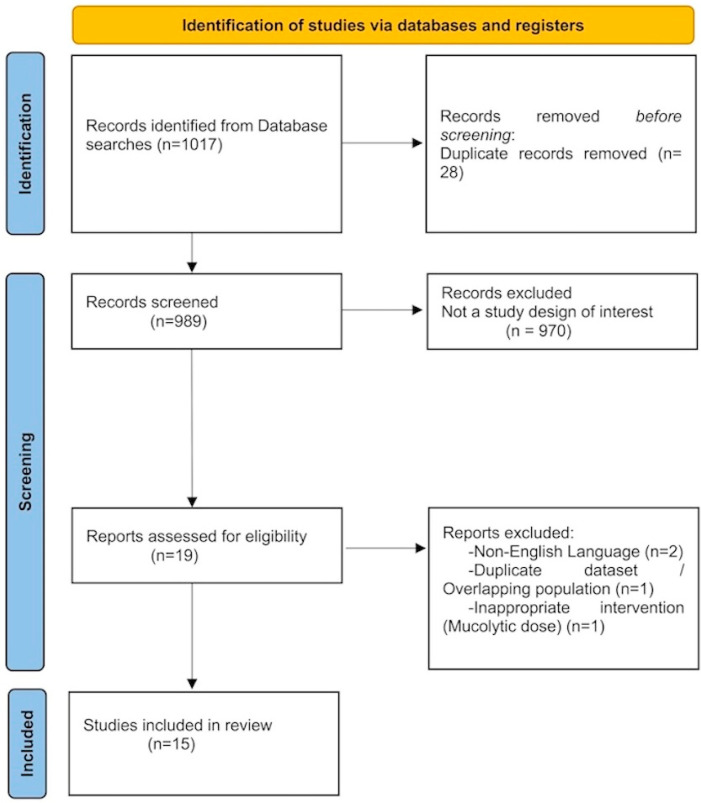
PRISMA Flow diagram of study selection process.

To capture every possible application of NAC in the context of TTP, we conducted searches across PubMed/MEDLINE, Google Scholar, and ClinicalTrials.gov for studies published up to January 2026 (Figure 1).

The detailed search strategy for PubMed/MEDLINE was as follows (“N-acetylcysteine” OR “N-acetyl-L-cysteine” OR “NAC”) AND (“Thrombotic Thrombocytopenic Purpura” OR “TTP” OR “Thrombotic Microangiopathy” OR “TMA” OR “Microangiopathic Hemolytic Anemia” OR “MAHA”). No restrictions were applied regarding study design. Filters were applied to include studies published up to January 2026 and limited to English language. Finally, acknowledging that the recent or niche data are often found in the “grey literature,” we rigorously hand-searched the reference lists of each relevant article and checked conference abstracts to ensure that small-scale case series were not overlooked. Google Scholar was used as a supplementary source to identify grey literature and additional records not indexed in major databases.

This systematic review was not prospectively registered in a public database (e.g., PROSPERO). While prospective registration is recommended to improve transparency and minimize reporting bias, the review was conducted based on predefined objectives, inclusion criteria, and outcomes. We acknowledge this as a limitation; however, we believe that it does not compromise the systematic approach or the validity of the findings.

### 2.2. Eligibility Criteria

We included studies involving patients (adult or child) with a confirmed diagnosis of immune-mediated or congenital TTP. The intervention of interest was NAC administration, whether used as a primary adjunctive therapy, a maintenance strategy, or a salvage therapy in resistant cases. Our analysis used the increase in platelet counts and the time to achieve complete clinical remission as criteria for clinical response. Mortality rates were also assessed as part of the follow-up. While we aimed for the highest level of evidence from randomized and cohort studies, the rare nature of TTP led us to include case reports, even those involving a single patient, to get a broader clinical picture. Duplicates, unrelated articles, review articles, and non-English studies were excluded. We followed the Preferred Reporting Items for Systematic Review and Meta-Analyses (PRISMA) guidelines to organize this systematic review [13]. The PRISMA checklists are provided as a Supplementary Files.

### 2.3. Data Extraction

To minimize bias, data extraction was performed by two independent observers. For each study meeting our criteria, we rigorously recorded specific NAC dosing protocols (including route and duration of administration) and the use of concomitant agents. Where precise treatment durations were not reported in the text, estimations were performed by two independent reviewers based on the provided clinical timelines and figures. In the event of disagreement between observers, a third senior hematologist was consulted to resolve discrepancies through consensus.

Considering the potential clinical heterogeneity and different sample sizes between studies, we decided that a qualitative narrative review would be more appropriate and clinically meaningful than a formal meta-analysis.

## 3. Results

### 3.1. Study Selection and Characteristics

A total of 1017 records were identified through database searching. After removing duplicate records (n = 28), the remaining 989 studies were screened by title and abstract. Of these, 970 records were excluded as they did not meet the study design criteria or were irrelevant to the topic. Upon full-text review of the remaining 19 studies, two case reports were excluded due to non-English language. One case report was excluded because the NAC therapy was administered at a mucolytic dose for respiratory symptoms. Another case report presenting two patients was excluded because the same patients were already included in a separate four-patient study incorporated into this review.

Consequently, 15 studies met the eligibility criteria and were included in the systematic review (Figure 1). The majority of the records were case reports or case series (n = 13), while two were larger observational studies (one cohort study and one observational case series). Detailed characteristics of the 15 included studies are presented in Table 1.

### 3.2. Patient Population and Demographics

A total of 70 patients treated with N-acetylcysteine (NAC) for TTP were analyzed across the 16 included studies. While the vast majority of patients were treated for acquired TTP, the review also captured rare etiologies. Specifically, Coşkun et al. [28] described a pediatric case of congenital TTP. The clinical setting for NAC initiation was predominantly in relapsed or refractory disease, although recent larger series have investigated its use in the acute phase alongside standard therapy.

### 3.3. NAC and Concomitant Therapies

Although there was heterogeneity in NAC treatment regimens across studies, the most frequently used dosage was 150 mg/kg or fixed doses roughly equivalent to this amount (e.g., 8–10 g/day). The timing of NAC initiation varied widely, occurring as late as day 135, particularly in refractory cases. In all cases, NAC therapy was administered as an adjunct to the standard treatment approach, which included Therapeutic Plasma Exchange (TPE), corticosteroids, and Rituximab. In some reports for patient’s refractory to this approach, additional agents, including vincristine, cyclophosphamide, and bortezomib, were added.

### 3.4. Clinical Outcomes

Time to platelet normalization (>150 × 10^9^/L) was highly variable across patients. In cases where NAC treatment was reported successful, platelet recovery was usually observed within 1 to 15 days after NAC initiation. The median time to platelet normalization in the case series by Español et al. [26] (n = 12) was reported as 6.5 days.

Thirteen of the 15 included studies reported complete response (CR), although definitions of response were not uniform. In one case, hematological remission was achieved without full neurological recovery, highlighting potential dissociation between hematologic and clinical outcomes.

A total of 10 deaths were reported in the largest cohort by Li et al. (2023) [27] (n = 38), representing the majority of fatal outcomes in this review. Additional isolated deaths included one case due to sepsis, and another associated with possible aortic dissection.

Importantly, the mortality findings from the Li et al. cohort contrast with the predominantly favorable outcomes reported in case reports and small series. Although the authors reported an association between NAC exposure and reduced mortality risk in multivariate Cox regression analysis (effect estimates not consistently reported), this finding should be interpreted with caution. As a single observational study, it is inherently subject to confounding factors, including disease severity, treatment selection bias, and timing of intervention. The discrepancy between crude mortality rates and adjusted outcomes further complicates interpretation.

When the studies were evaluated according to clinical context, NAC appeared to be more frequently used in relapsed or refractory TTP, where platelet responses were often reported within a relatively short timeframe. In contrast, in studies where NAC was initiated earlier in the disease course, the contribution of NAC to clinical improvement was more difficult to isolate due to concomitant standard therapies.

Furthermore, variability in response patterns was notable across studies. While several reports described rapid platelet recovery following NAC initiation, others demonstrated partial or delayed responses, suggesting that the effectiveness of NAC may be influenced by disease severity, timing of initiation, and concurrent immunosuppressive treatments.

### 3.5. Risk of Bias and Quality Assessment

The methodological quality of the reviewed articles is summarized in Table 2. Since our analysis included a variety of study designs—ranging from single-case reports to cohort studies—applying a single standard checklist was not feasible. Instead, we adapted criteria from the Joanna Briggs Institute (JBI) critical appraisal tools to create a unified scoring system [29]. Each study was evaluated across five main areas: how well the patients were described, the certainty of the diagnosis, the clarity of the intervention, the reporting of clinical outcomes, and the length of follow-up. We rated the provided data in each domain as ‘Adequate’, ‘Partial’, or ‘Limited’. Ultimately, the accumulation of these individual scores determined whether a paper’s overall risk of bias was categorized as Low, Moderate, or High.

Based on this unified evaluation, the methodological quality of the reviewed articles is generally moderate. Since the available data predominantly comes from single-case reports and small series, a certain degree of inherent bias is unavoidable. Most authors did an excellent job reporting specific intervention details and confirming the ADAMTS13-based diagnosis. However, significant limitations emerged regarding short or poorly detailed follow-up periods, alongside the expected lack of control groups and small sample sizes. Consequently, due to these accumulated ‘Partial’ and ‘Limited’ domain scores, the majority of the included studies are considered to carry a moderate to high overall risk of bias.

## 4. Discussion

In this systematic review, we compiled the current clinical evidence regarding the use of NAC in the treatment of TTP and found NAC use in the literature across 16 studies involving 70 patients. It appears to be used primarily as an adjunct therapy in relapsed or refractory cases; however, more recent observational data also show uses in the acute phase. Although many articles have highlighted associations between NAC initiation and platelet count improvement, the overall quality and consistency of the evidence is limited. When examined collectively, the available evidence suggests a heterogeneous but potentially clinically meaningful pattern: NAC may contribute to platelet recovery, particularly in refractory settings; however, its independent therapeutic effect remains difficult to delineate due to the consistent use of combination therapies. The current treatment of the iTTP mainly consists of TPE to replenish the patient’s plasma with ADATMS13 and immunosuppressants and steroids to downregulate antibody production. Caplacizumab distinguishes itself from current therapies by inhibiting the interaction between vWF and platelets, thereby targeting a distinct pathological pathway. Similarly, NAC provides a unique pharmacokinetic advantage by reducing ultra-large vWF multimers through the disruption of disulfide bonds. In the literature, NAC is predominantly utilized in refractory cases or resource-limited settings, acting as a synergistic adjunct to standard therapeutic lines.

The most striking feature of the current literature is its significant heterogeneity. It should be emphasized that, most of the included studies are single-case reports or small case series showing considerable variability in patient characteristics, disease stage (newly diagnosed vs. resistant), ADAMTS13 status reporting, concomitant therapies, NAC dosing regimens, and timing of initiation. In most cases, NAC has been administered in conjunction with therapeutic plasma exchange, corticosteroids, rituximab, and in some cases additional immunosuppressive agents. For these reasons, it is difficult to evaluate the independent therapeutic contribution of NAC. Data on optimal timing are also variable, preventing a clear conclusion from being drawn.

Another point to highlight is the difference in results between smaller descriptive articles and larger observational cohorts. Case reports and small series describe markedly positive hematological responses after NAC administration. However, the largest cohort included in this review reports a notable mortality rate, although multivariate analysis suggests an association between NAC exposure and reduced hospital mortality. This divergence between highly favorable outcomes in case reports and more modest results in larger observational cohorts suggests the presence of publication bias and underscores the need for cautious interpretation.

Dosage strategies represent one of the most significant differences in the reports. While many studies have used fixed daily doses of approximately 150 mg/kg/day or 8–10 g, there is no standard protocol regarding loading strategies, maintenance duration, or treatment discontinuation criteria. Variability in platelet normalization time is present in the studies and does not allow for the establishment of a clear dose–response relationship. Without pharmacokinetic and pharmacodynamic correlation studies evaluating VWF multimer dynamics and ADAMTS13 recovery in vivo, the mechanistic effect of NAC in a clinical setting remains inferential. Intravenous NAC is associated with well-described anaphylactoid reactions, which are typically non–IgE-mediated histamine release and occur early during infusion, with reported incidence ranging from 8% to nearly 44% depending on patient characteristics and study design. However, most of the data relates to the use of NAC due to paracetamol toxicity [30]. Again, when evaluated in terms of reliability, clinically significant drug–drug interactions of N-acetylcysteine are relatively uncommon; However, its thiol-donating and antioxidant properties may lead to pharmacodynamic interactions through redox modulation and direct chemical binding, particularly enhancing nitric oxide–mediated vasodilatory responses when co-administered with organic nitrates [31]. Furthermore, safety reporting in studies is limited. The literature reviewed did not reveal consistent evidence of serious toxicity attributable to NAC. Data on NAC use in TTP do not mention any findings related to drug interactions or anaphylactoid reactions. Given its widespread use in other clinical settings and its well-known safety profile, NAC appears to be tolerable in TTP patients. In addition to these, from a healthcare system perspective, NAC’s accessibility and low cost distinguish it from existing targeted biological drugs. In resource-constrained settings where advanced therapies are unavailable or unaffordable, NAC potentially offers a practical adjunctive therapy option.

In addition to the limitations of the included studies, our study also has methodological limitations. The search strategy was limited to three databases, and non-English publications were excluded; this may have led to the omission of relevant reports. Furthermore, the weighting of case reports may have resulted in a higher likelihood of reporting successful or unusual cases, leading to publication bias. Another limitation is the lack of prospective protocol registration, which may increase the risk of reporting bias. Also, the predominance of case reports and small observational series limits the strength of the available evidence and increases the risk of bias, which should be considered when interpreting the findings.

Based on the available data, it is undeniable that well-designed prospective studies are needed. Standardized dosage protocols, detailed response criteria, relapse outcome results, and detailed safety assessments are necessary to clarify whether NAC provides an additional benefit among current treatment strategies. Randomized controlled trials comparing NAC with and without standard care are needed.

## 5. Conclusions

Current findings suggest that NAC may have a biologically rational and potentially adjunctive value in TTP, particularly in resistant disease or when access to costly targeted therapies is limited. However, the current literature is limited to heterogeneous case reports and small observational studies, preventing definitive conclusions on efficacy.

While the literature data is encouraging, it is debatable whether it is sufficient to support the routine inclusion of NAC in standard treatment algorithms. Currently, NAC should be considered a promising but unproven adjunctive therapy. Its favourable safety profile, global availability, and low cost increase its feasibility.

Most importantly, future randomized controlled trials are needed to determine whether NAC significantly improves clinically relevant outcomes, including platelet recovery time, relapse rates, and mortality, when added to current standard treatment.

## Figures and Tables

**Table 1:** Clinical Data of Studies Using N-Acetyl Cysteine in Thrombotic Thrombocytopenic Purpura Patients.

First Author/Year	NAC-Treated Patients (n)	NAC Dosage/ Regimen	NAC Administration (Start Day/Duration)	Time to Platelet Normalization (Days from Admission)	Clinical Response/(Follow-Up If Reported)	Additional Treatments
Chapin et al., 2011 [14]	1	NR	Day 13/8 days *	56	CR, Relapse (Day 85)	MP, PEX, RTX, VCR, Eculizumab
Shortt et al., 2013 [15]	1	2.5 g/day	Day 14/14 days *	56	CR, (Relapse NR)	MP, PEX, RTX, CYC, BTZ
Li et al., 2014 [16]	1	150 mg/kg/day	Day 13/10 days	23	CR, (Relapse NR)	MP, PEX, RTX
Chen et al., 2015 [17]	2	150 mg/kg/day	Day 2/4 days	1 Day After NAC Therapy	CR, (Relapse NR)	MP, (Other treatments are NR)
Cabanillas & Popescu-Martinez, 2015 [18]	1	150 mg/kg/day	Day 135/10 days	NR	CR, (Relapse NR)	MP, PEX, RTX, CYC, VCR
Rottenstreich et al., 2016 [19]	3	300/kg/day	Day 10, Day 1, Day 2/4 days, 9 days, 7 days *	15, 3, 8	CR, (6, 30, 6 months)	MP, PEX, RTX, VCR, Dexamethasone
Acedillo et al., 2016 [20]	1	18 g/day	Day 21/9 days	80	CR with neurological deficits, (1 year)	MP, PEX, RTX, CYC, BTZ
Patriquin et al., 2016 ^†^ [21]	1	NR	Day 10/4 days	31	CR, (Relapse NR)	MP, PEX, RTX, BTZ, MMF
Demircioğlu et al., 2018 [22]	1	150 mg/kg/day (10 g/day)	Day 36/5 days	41	CR, (6 months)	MP, PEX, RTX, VCR
Azapağası et al., 2020 [23]	1	100 mg/kg/day	Day 1/90 days	90	CR, (Relapse NR)	MP, PEX, RTX, CYC, BTZ
Huang et al., 2021 [24]	1	NR	Day 5/17 days	35	CR, (3 months later died of possible dissection)	MP, PEX, RTX
Beyler & Demir, 2023 [25]	4	10 g/day	Day 10, Day 12, Day 10, Day 30 */10 days, 10 days, NR, 5 days	1, 4, 2, 5 (Days After NAC Therapy)	CR, (3, 6, 2, 6 months)	MP, PEX, RTX, VCR
Español et al., 2023 [26]	12	150 mg/kg/day	Day 2 (Median) ^§^/10 days	6,5 Days (Median)	CR (29 months median follow-up, 2 relapse)	MP, PEX, RTX, BTZ, Mycophenolate Mofetil
Li et al., 2023 [27]	38	8 g/day	7–14 days	7 Days (Median)	28 Response, 10 Deaths	MP, PEX, RTX
Coşkun et al., 2025 [28]	1	NR	Day 3/11 days	5	CR (3 episodes of mild TMA in 4 years)	PEX, FFP, Factor VIII
Total	69					

Platelet normalization was defined by a platelet count above 150 × 109/L. Abbreviations: NAC, N-Acetyl Cysteine; NR, Not Reported; CR, Complete Response; PEX, Plasma Exchange; RTX, Rituximab; MP, Methylprednisolone; CYC, Cyclophosphamide; VCR, Vincristine; BTZ, Bortezomib. * Specific data not explicitly provided in the text; estimated based on the clinical figure and text-based milestones. ^†^ Included the single patient treated with NAC out of 6 reported cases. ^§^ NAC was started on the 12th day in the first case, on the 76th day in the eighth case, and on the second day in the other 10 cases.

**Table 2:** Risk of Bias Assessment of Included Studies.

Study	Design	Patient Description	Diagnostic Confirmation	Intervention Clarity	Outcome Reporting	Follow-Up Adequacy	Overall Risk of Bias
Chapin et al., [14]	Case report	Adequate	Confirmed	Partial	Adequate	Adequate	Moderate
Shortt et al., [15]	Case report	Adequate	Confirmed	Adequate	Adequate	Limited	Moderate
Li et al., [16]	Case report	Adequate	Confirmed	Adequate	Adequate	Limited	Moderate
Chen et al., [17]	Case series (n = 2)	Adequate	Confirmed	Adequate	Adequate	Limited	Moderate
Cabanillas & Popescu-Martinez, [18]	Case report	Adequate	Probable	Adequate	Adequate	Limited	Moderate
Rottenstreich et al., [19]	Case series (n = 3)	Adequate	Mixed (confirmed/probable)	Adequate	Partial	Limited	Moderate-High
Acedillo et al., [20]	Case report	Limited	Probable	Adequate	Partial	Limited	High
Patriquin et al., [21]	Case series (Total n = 6, *NAC n = 1)	Adequate	Confirmed	Partial	Adequate	Adequate	Moderate
Demircioğlu et al., [22]	Case report	Adequate	Confirmed	Adequate	Adequate	Adequate	Low-Moderate
Azapağası et al., [23]	Case report	Adequate	Confirmed	Adequate	Adequate	Adequate	Low-Moderate
Huang et al., [24]	Case report	Limited	Probable	Partial	Partial	Limited	High
Beyler & Demir, [25]	Case series (n = 4)	Adequate	Confirmed	Adequate	Adequate	Limited	Moderate
Español et al., [26]	Observational case series (n = 12)	Adequate	Confirmed	Adequate	Adequate	Adequate	Low-Moderate
Li et al., [27]	Retrospective cohort study (Total n = 89, NAC n = 38)	Adequate	Confirmed	Adequate	Adequate	Adequate	Low-Moderate
Coşkun et al., [28]	Case report	Adequate	Confirmed	Partial	Adequate	Adequate	Moderate

Risk of bias was assessed using adapted criteria based on the Joanna Briggs Institute (JBI) critical appraisal tools for case reports, case series and cohort studies. Studies were evaluated across domains including patient description, diagnostic confirmation, intervention clarity, outcome reporting, and adequacy of follow-up. *NAC, N-Acetyl Cysteine.

## Data Availability

No new data were created or analyzed in this study.

## Supplementary Materials

The following supporting information can be downloaded at: https://www.mdpi.com/article/10.3390/jcm15072713/s1, File S1: PRISMA 2020 checklist; File S2: PRISMA 2020 abstract checklist.

## Abbreviations

MHA	Microangiopathic Hemolytic Anemia.
NAC	N-acetylcysteine
PRISMA	Preferred Reporting Items for Systematic Review and Meta-Analyses
TMA	Thrombotic Microangiopathy
TPE	Therapeutic Plasma Exchange
TTP	Thrombotic Thrombocytopenic Purpura
ULVWF	Ultra-Large von Willebrand Factor
vWF	von Willebrand Factor
